# Repeated bouts of exhaustive exercise increase circulating cell free nuclear and mitochondrial DNA without development of tolerance in healthy men

**DOI:** 10.1371/journal.pone.0178216

**Published:** 2017-05-24

**Authors:** Robert Stawski, Konrad Walczak, Piotr Kosielski, Pawel Meissner, Tomasz Budlewski, Gianluca Padula, Dariusz Nowak

**Affiliations:** 1 Department of Clinical Physiology, Medical University of Lodz, Lodz, Poland; 2 Department of Internal Medicine and Nephrodiabetology, Medical University of Lodz, Lodz, Poland; 3 Academic Laboratory of Movement and Human Physical Performance, Medical University of Lodz, Lodz, Poland; 4 Department of Internal Medicine, University Hospital name of the Military Medical Academy—Central Hospital Veterans of Lodz, Lodz, Poland; Kaohsiung Medical University, TAIWAN

## Abstract

**Objective:**

Acute single strenuous exercise increases circulating cell free DNA (cf DNA). We tested whether three repeated bouts of exhaustive exercise induced the cf DNA response without development of tolerance in healthy men.

**Methods:**

Eleven average-trained men (age 34.0±5.2 years, body mass index 26.2±3.1 kg/m2, maximal oxygen consumption—VO_2_max 49.6±4.5 ml/kg*min) performed three treadmill exercise tests to exhaustion at speed corresponding to 70% VO_2_max separated by 72 hours of resting. Blood was collected before and after each bout of exercise for determination of cell free nuclear and mitochondrial DNA (cf n-DNA, cf mt-DNA) by real-time PCR, selected markers of muscle damage, and blood cell count.

**Results:**

Each bout induced the increase (p<0.05) in plasma cf n-DNA: from 3.4±1.4 to 38.5±27.5, from 4.1±3.3 to 48.5±26.2, and 3.1±1.6 to 53.8±39.9 ng/mL after the first, second, and third exercise, respectively. In a congruent way, cf mt-DNA rose significantly after the second (from 229±216 to 450±228*103 GE/mL) and third bout of exercise (from 173±120 to 462±314*103 GE/mL).

Pre-exercise cf mt-DNA decreased (p<0.05) by 2-times (from 355±219 before the first bout to 173±120*103 GE/mL before the third bout) over the study period and were accompanied by significant increase in white blood cells, platelets, creatine kinase, creatinine and lactate after each bout. However, the exercise induced percentage increment of cf n-DNA was always many times higher than corresponding increments of the afore-mentioned markers at any occasion.

**Conclusions:**

Repeated bouts of exhaustive exercise induced remarkable increase in circulating cf n-DNA without signs of tolerance development. Baseline cf mt-DNA decreased in response to series of strenuous exercise. Since percentage increments of cf n-DNA in response to exercise were many times higher than those observed for other markers, measurement of circulating cf n-DNA could be a sensitive tool for monitoring acute exercise effects in human body.

## Introduction

Repeated bouts of exercise are an effective impulse for physiological adaptation. Skeletal muscle reveals notable plasticity in functional adaptation and remodeling in response to acute or chronic physical activity. Training-induced adaptations are reflected by changes in the contractile proteins, mitochondrial function, metabolic regulation, intracellular signaling, and transcriptional response [1]. Prolonged endurance training elicits a variety of metabolic and morphological changes, including mitochondrial biogenesis, muscle fiber type transformation, increased capilarization, improved regulation of K+, H+ and lactate ions, and more efficient substrate metabolism [2, 3]. However, extensive training can also be accompanied by some adverse effects associated with the inflammatory response. Lactic acid accumulation and damage to muscle fibers may induce the rise in plasma activities of certain enzymes including creatine kinase (CK), asparate aminotransferase (AST) and alanine aminotransferase (ALT), as well as the elevation of circulating cell free DNA [4, 5]. Cell free DNA (cf DNA) can be divided in two polls according to its origin: cell free nuclear DNA (cf n-DNA) and cell free mitochondrial DNA (cf mt-DNA), deriving from nucleus or cytoplasmic mitochondria’s, respectively. Increased levels of circulating cf n-DNA were observed under variety of pathological conditions including cancer, autoimmune diseases, stroke, traumatic brain, and other organs injury [6–10]. In contrast to the afore-mentioned pathological conditions, exercise-induced increase in cf n-DNA is transient [11–13], probably due to the parallel elevation of serum DNAse 1 activity leading to its normalization within 0.5 to 2 hours of recovery [13]. Moreover, exercise-induced increase in cf n-DNA is not specific to sport disciplines, however, seems to be strictly related to the load of physical exertion [7–14]. On the other hand, studies on the association between exercise and circulating levels of cf mt-DNA gave controversial results. Exhaustive exercises related to 10 km cross-country run and treadmill running workout did not alter plasma cf mt-DNA levels [15,16], while in other trials involving treadmill runners and professional male volleyball players, exercise surprisingly caused the decline in cf mt-DNA [17]. Although the cellular source of exercise-induced cf DNA is still an unresolved question (apoptotic or necrotic cells, skeletal muscles, blood mononuclear cells, cells of the hematopoietic lineage) [18,19], this phenomenon is supposed to be the predictor of muscle damage, immunological alterations, inflammatory response, and even the overtraining syndrome in elite athletes [6–14]. However, the intensity of cf DNA response to the repeated bouts of strenuous exercise has not been studied so far. One may suppose that due to some adaptive processes these cf DNA responses would decline in the order of appearance. Therefore, in this study we wanted to determine the effect of three repeated exhaustive treadmill runs (separated each by 3 days of recovery) on cf n-DNA and cf mt-DNA plasma levels in healthy physically active young men. Furthermore, the intensity of cf DNA response was compared with the exercise-induced rise in white blood cells (WBC), circulating levels of lactic acid, creatinine and activities of AST, ALT and CK.

## Materials and methods

### Studied population

The study included eleven apparently healthy, average-trained men [mean age 34.0±5.2 years, mean body weight 87.4±13.8 kg, mean body mass index 26.2±3.1 kg/m^2^, maximal oxygen consumption (VO_2_ max) 49.6±4.5 ml/kg*min, forced vital capacity (FVC) 6.09±0.41 L, 106.4±6.4% of predicted, forced exhaled volume in the first second (FEV_1_) 4.93±0.45 L, FEV1/FVC 80.9±5,6%] members of the medical university of Lodz, Poland.

The inclusion criteria were age between 25 and 45 years, a written informed consent before initiating the study procedures. The exclusion criteria included alcohol and illicit drug abuse, current cigarette smoking, any history of infectious or inflammatory diseases, use of any vitamins or food supplements, or any systemic pharmacological treatment within 3 months prior to the study. All subjects agreed to keep their dietary habits constant during the study period and to comply with the instructions related to participation in the study.

### Study protocol

**Fig 1:**
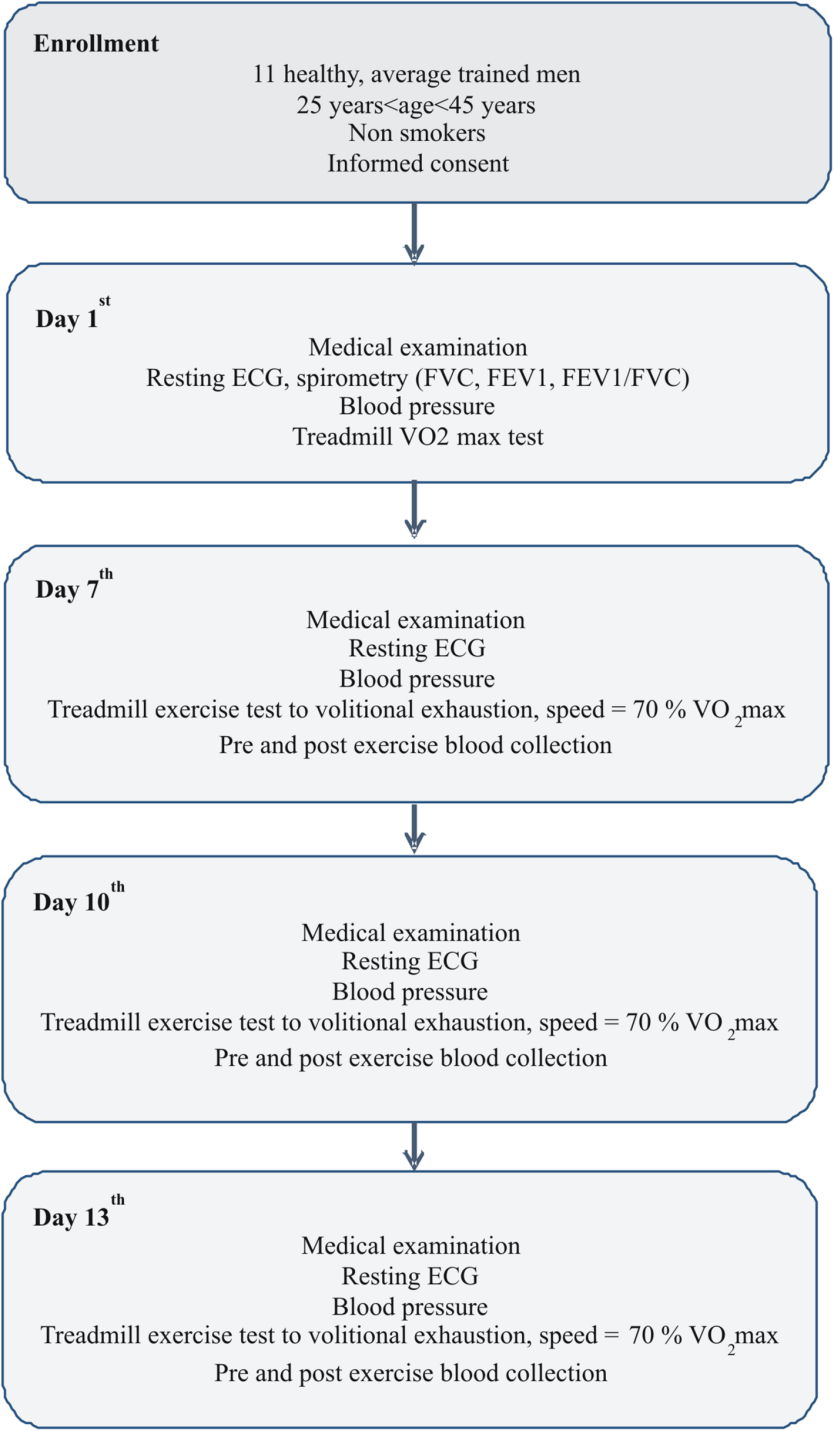
Study design flowchart. ECG -electrocardiography, VO_2_ max—maximal oxygen consumption, FVC—forced vital capacity, FEV_1_—forced exhaled volume in the first second. During the whole study period (13 days), volunteers did not perform any exhaustive exercise besides those related to the study protocol.

The study consisted of 4 visits at the 1^st^, 7^th^, 10^th^ and 13^th^ day of observation (Fig 1).

All visits started at 9:00 AM and included medical examination, blood pressure measurement, and resting electrocardiography to exclude any contraindications to exercise test. At the first visit (day 1^st^) subjects underwent spirometry tests (measurement of FVC, FEV_1_ and FEV_1_/FVC ratio) and treadmill VO_2_ max test. Afterwards, at the three consecutive visits (day 7^th^, 10^th^, and 13^th^) participants performed treadmill exercise to volitional exhaustion at speed corresponding to 70% of their personal VO_2_ max. Pre- and immediately post-exhaustive exercise venous blood samples (15 ml) were collected into vacutainer tubes (Becton Dickinson, Franklin Lakes, NJ) with EDTA for cell free DNA extraction and blood cell count, into tubes containing gel and clot activator for blood chemistry, and into tubes with sodium oxalate and potassium fluoride for lactate determination. All exercise bouts were performed at the Academic Laboratory of Movement and Human Physical Performance “DynamoLab” of the Medical University of Lodz at ambient temperature 20–21°C and relative air humidity 50–60%. During the whole study period (13 days) volunteers did not perform any exhaustive exercise besides those related to study protocol. The study was conducted according to the Declaration of Helsinki. The protocol was reviewed and approved by The Medical University of Lodz Ethics Committee (RNN/95/14/KB), and all volunteers provided written informed consent.

### Determination of VO_2_ max

VO_2_ max was determined by a continuous incremental maximal exercise test on a programmable treadmill (Trackmaster CP 425) interfaced with a Ultima CardiO_2_ PFX (gas exchange analysis system) integrated with a 12-lead wireless Mortara ECG (Medical Graphics Corporation, St Paul, MN, USA). After a short warm up (3 minutes of walking at their self-selected pace and 5 minutes of jogging at a speed of 6 km/h), subjects started to run with initial speed 7 km/h and constant treadmill inclination of 1.5%. Speed was increased every 3 minutes by 1.5 km/h (incline was left unchanged at 1.5%) until volitional exhaustion defined as subjects inability to maintain the required exercise intensity or their wish to stop the test. In all cases the following three criteria had to be met to determine the VO_2_ max: (a) plateau in the oxygen consumption despite an increase in running speed, (b) respiratory exchange ratio >1.10, and (c) peak heart rate exceeding 90% of age predicted maximum (220-age) [20]. Not more than one subject was examined per day, and the gas exchange analysis system was calibrated before each exercise test according to manufacturer instruction.

### Execution of treadmill exhaustive exercise

All subjects had usual breakfast between 7:00 and 8:00 AM. At 10:00 AM (after all physical exams and exclusion of contraindications to exercise testing) subjects started to run on the treadmill (Trackmaster CP425) with a speed corresponding to 70% of their personal VO_2_ max and at a constant treadmill inclination of 1.5% until volitional exhaustion. Heart rate was monitored with the Polar chest strap H7 heart rate sensor. Subjects were allowed to only drink mineral water during exercise. Body weight and blood pressure were measured as well as venous blood samples were taken before and just after exhaustive treadmill run. A separate written informed consent for each of 3 exhaustive bouts was obtained from all studied volunteers.

### Blood sample processing and cell free DNA extraction

Plasma from EDTA blood samples was obtained by centrifugation (1600 x g, 4°C) for 10 min. Then, plasma samples were subjected to the second centrifugation (16 000 x g, 4°C, 5 min) to remove the cell debris and were stored at -80°C for no longer than 4 weeks until concentration of cf DNA measurements were obtained. Cell free DNA (cf n-DNA and cf mt-DNA) was isolated from 400 μL plasma using a QIAamp DNA Blood Mini Kit (Qiagen GmbH, Hilden, Germany) according to manufacturer instructions with elution into 40 μL TE buffer.

### Real-time PCR for the measurement of cf n-DNA and cf mt-DNA in plasma

For the quantification of isolated from plasma cf n-DNA and cf mt-DNA a quantitative real-time PCR (qPCR) was performed, in Central Scientific Laboratory “CoreLab” of the Medical University of Lodz, using the glyceraldehyde 3-phosphate-dehydrogenase (GAPDH) gene for cf n-DNA and mitochondrially encoded ATP synthase 8 (MT-ATP 8) gene for the cf mt-DNA according to the method described previously [8, 21]. The primer sequence used for testing the GAPDH gene was 5’-CCCCACACACATGCACTTACC-3’ for the forward primer and 5’-CCTAGTCCCAGGGCTTTGATT-3’ for the reverse primer and 5’-MGB-TAGGAAGGACAGGCAAC–VIC-3’ as the probe. In the case of MT-ATP 8 gene, the sequences of the forward primer, reverse primer and the probe were 5’AATATTAAACACAAACTACCACCTACC-3’, 5’-TGGTTCTCAGGGTTTGTTATAA-3’ and 5’-FAM-CCTCACCAAAGCCCATA-MGB-3’, respectively. Simultaneous multiplex qPCR was carried out in 20 μL of total reaction volume containing 5 μL H_2_O, 10 μL TaqMan® Universal PCR Master Mix (Applied Biosystems, Branchburg, New Jersey, USA), 0.25 μL of each of the four afore-mentioned primers (Sigma-Aldrich), 1 μL of a FAM-labeled MT-ATP 8-probe, 1 μL of a MVIC-labeled GAPDH-probe (both probes from Applied Biosystems), and 2 μL of TE buffer containing cf DNA isolated from plasma. The final concentrations of primers and probes were: 0.6 μmol/L and 0.4 μmol/L, respectively. Negative control samples received 2 μL of TE buffer without cf DNA from plasma. Reaction was done in duplicate and performed using the 7900 HT Real-time PCR System (Applied Biosystems) under the following conditions: an initiation step at 50°C for 2 min, followed by a first denaturation at 95°C for 10 min, then 40 cycles of 95°C for 15 s and annealing at 60°C for 1 min.

Serial dilutions of human genomic DNA (Roche) (final concentrations from 0.5 ng/mL to 5000 ng/mL) were used to construct the calibration curves for cf n-DNA and cf mt-DNA measurement with r^2^ = 0.9659 and r^2^ = 0.9996, respectively. Individual results were obtained as a mean from two separate runs and expressed in ng/mL for cf n-DNA and as genome equivalents (GE)/ml plasma (1 GE = 6.6 pg DNA) for cf mt-DNA [8, 21].

### Other determinations

Complete blood count, serum activities of creatine kinase (CK), asparate aminotransferase (AST), alanine aminotransferase (ALT), and concentrations of C-reactive protein (CRP), lactic acid, glucose, urea, and creatinine were determined in the Diagnostic Laboratory of Central Clinical Hospital of the Medical University in Lodz.

### Statistical analysis

Results are expressed as a mean (SD) and median. Analysis of variance (ANOVA) for repeated observations or Friedman’s ANOVA was applied for the assessment of changes in variables over time (before and after three consecutive exercise bouts) depending on data distribution which was tested with Shapiro-Wilk’s W test. In case of statistically significant ANOVA, the post hoc analyses were done with Scheffe’s test or post hoc analysis for Friedman’s ANOVA (multiple comparisons at 2 different time points). For comparison of magnitude changes of variables related to consecutive exercise bouts, the percentage increments were calculated according to the formula: (value after bout–value before bout)/value before bout] x 100%, and analyzed as repeated observations. Comparisons of exercise induced–percentage increments of two given variables (e.g. cf n-DNA vs CK) were analyzed with the Mann–Whitney *U* test. A p value<0.05 was considered significant.

## Results

### Characteristics of exhaustive treadmill exercise bouts

All included men (n = 11) successfully completed the study protocol of three repeated exhaustive treadmill exercises executed every three days. Table 1 shows additional subjects characteristics and selected parameters related to exercise tests including run distance to exhaustion, loss of body mass, and maximal heart rate reached during the test. All these parameters were similar for each of the three bouts except the shorter run distance to exhaustion at the first bout than those reached at the remaining two ones (p< 0.05). Although, subjects were allowed to drink mineral water during the bouts, exhaustive exercise resulted in the decrease in mean body mass by about 0.7 to 1 kg (Table 1). Corresponding to this, hematocrit, hemoglobin levels, and the number of erythrocytes revealed the tendency to increase in response to exercise (Table 2) what could be the result of dehydration. Therefore, all results obtained from plasma or serum analysis were corrected for hematocrit shift related to exercise-induced water loss. White blood cell count and the number of granulocytes, lymphocytes, monocytes, and platelets raised significantly after each exercise bout, however, baseline values (before exercise) did not differ from each other (Table 2).

**Table 1:** Parameters monitored during three repeated exhaustive treadmill exercise bouts separated by three days of resting period.

Parameter	Bouts of exhaustive treadmill exercise		
	1^st^ bout–day 7	2^nd^ bout–day 10	3^rd^ bout–day 13
Run distance to exhaustion [km]	8.6±5.5	10.7±7.6*	10.4±7.2*
Run time [min]	47±31	57±41	56±40
Baseline heart rate [beats/min]	72±11	76±12	69±9
Maximal heart rate [beats min]	184±10	183±11	176±12
Baseline blood pressure [mm Hg] S/D	127±6 / 80±4	128±7 / 80±6	122±9 / 74±5
Blood pressure after exercise [mm Hg] S/D	172±20 / 82±11	169±13 / 80±10	166±13 / 79±11
Loss of body mass [kg]	0.73±0.65	0.84±0.81	1.05±0.78

S–systolic, D–diastolic. Eleven average-trained men completed the study composed of four visits at days 1, 7, 10 and 13. After determination of VO_2_ max at day 1, three repeated treadmill exercise tests to exhaustion at speed corresponding to 70% of personal VO_2_ max were executed at days 7, 10 and 13. Results are expressed as mean ±SD.

* vs corresponding value at 1^st^ bout, p<0.05

**Table 2:** Blood cell count in eleven average-trained men before and after each of three bouts of exhaustive treadmill exercise.

Blood cell count	Bouts of exhaustive treadmill exercise					
	1^st^ bout		2^nd^ bout		3^rd^ bout	
	before	after	before	after	before	after
Hct [%]	46.5±3.4 (48.6)	48.1±3.4 (48.1)	45.9±3.6 (46.5)	47.8±3.6 (48.6)	44.1±3.9 (45.2)	46.7±3.9 (46.4)
Hgb [g/dl]	15.6±1.1 (16.0)	16.1±1.2 (15.9)	15.3±1.1 (15.5)	15.9±1.1 (16.1)	14.8±1.3 (15.2)	15.5±1.2 (15.7)
RBC [x10^6^/mm^3^]	5.17±0.41 (5.08)	5.32±0.42 (5.23)	5.09±0.48 (5.04)	5.28±0.46 (5.24)	4.89±0.51 (4.95)	5.16±0.50 (5.19)
WBC [x10^3^/mm^3^]	5.86±0.62 (5.61)	9.45±1.94* (9.32)	5.68±0.59 (5.60)	9.99±2.87* (8.65)	5.76±0.49 (5.73)	10.7±3.24* (9.51)
GRA [x10^3^/mm^3^]	3.98±0.71 (3.91)	6.02±1.88* (5.43)	3.85±0.68 (3.82)	6.46±2.92* (5.46)	3.97±0.60 (3.84)	7.27±3.49* (6.03)
MON [x10^3^/mm^3^]	0.20±0.04 (0.21)	0.36±0.08* (0.42)	0.19±0.05 (0.23)	0.38±0.10* (0.43)	0.22±0.04 (0.20)	0.40±0.10* (0.41)
LYM [x10^3^/mm^3^]	1.68±0.33 (1.63)	3.07±0.81* (2.91)	1.64±0.30 (1.64)	3.15±0.78* (3.05)	1.57±0.29 (1.60)	3.05±0.42* (2.96)
PLT [10^3^/mm^3^]	208±33 (199)	258±40* (248)	200±28 (209)	248±32* (255)	199±34 (206)	247±44* (258)

Hct–hematocrit, Hgb–hemoglobin, RBC–red blood cells, WBC–white blood cells, GRA–granulocytes, MON–monocytes, LYM–lymphocytes, PLT–platelets. Results are expressed as mean ± standard deviation (and median in parentheses). Other details as for Table 1.

* vs corresponding value before the bout, p<0.05

### Changes of selected markers of muscle damage and metabolic response to three repeated bouts of exhaustive treadmill exercise

Significant increase (p<0.05) in circulating CK was observed after each session of exhaustive exercise (Table 3). There was no normalization of CK during 72 hours of resting period between repeated bouts. Mean CK levels after the first bout and before the second one as well as after the second and before the third ones were similar. Consequently, baseline CK values (before the bout) revealed significant trend to increase in order of measurement (162±63 U/L vs 267±216 U/L vs 301±131 U/L, p<0.05). AST and ALT behaved similarly: significant increase in response to the third bout of exercise and no differences between corresponding values observed before and after the first and second ones. Concentration of lactate and creatinine raised significantly after each exhaustive exercise, however, baseline values of these variables did not differ from each other. Similarly, urea, glucose, and C-reactive protein levels tended to increase after each bout of exercise, however, the significant difference (p<0.05) was observed only after the first one (Table 3). Mean pre-exercise C-reactive protein levels revealed the moderate upward trend over the study period.

**Table 3:** Selected markers of muscle damage and metabolic response to exercise before and after each of three bouts of exhaustive treadmill exercise.

Marker	Bouts of exhaustive treadmill exercise					
	1^st^ bout		2^nd^ bout		3^rd^ bout	
	before	after	before	after	before	After
CK (U/l)	162±63 (141)	211±112* (179)	267±216 (158)	302±184* (197)	301±131 (299)	348±130*† (320)
AST (U/l)	26±7 (25)	35±19 (30)	29±8 (27)	36±17 (29)	32±10 (33)	54±68* (31)
ALT (U/l)	28±8 (26)	33±14 (28)	29±10 (28)	33±12 (31)	32±11 (35)	48±52* (35)
Lactate (mmol/l)	1.7±0.8 (2.1)	8.9±4.6* (8.0)	1.4±0.7 (1.2)	8.8±5.0* (9.9)	1.6±0.5 (1.5)	8.0±4.5* (7.6)
Creatinine (μmol/l)	84.8±11.6 (81.0)	116.0±6.8* (112.1)	84.3±10.0 (83.2)	113.5±11.1* (106.5)	85.8±13.8 (80.4)	105.9±12.6* (94.1)
Urea (mmol/l)	5.8±1.2 (5.6)	6.4±1.5* (5.8)	5.7±1.1 (5.7)	6.4±1.4 (5.7)	6.1±1.7 (5.5)	6.7±1.7 (5.5)
Glucose (mmol/l)	5.2±0.8 (5.2)	6.8±1.8* (6.1)	5.9±1.1 (5.6)	6.9±1.9 (5.9)	5.1±1.0 (5.1)	6.3±1.8 (5.6)
CRP [mg/L]	0.99±0.60 (0.80)	1.53±1.28* (1.17)	1.29±1.10 (1.00)	1.31±1.29 (0.71)	1.73±1.76 (1.00)	1.92±1.91 (1.04)

CK–creatine kinase, AST–asparate aminotransferase, ALT–alanine aminotransferase, CRP–C-reactive protein. Results are expressed as mean ± standard deviation (and median in parentheses). Other details as for Table 1.

* vs corresponding value before the bout, p<0.05.

† vs corresponding values before the first and the second bout, p<0.05.

### Changes of plasma concentrations of cell free nuclear and mitochondrial DNA in response to three repeated bouts of exhaustive treadmill exercise

**Fig 2:**
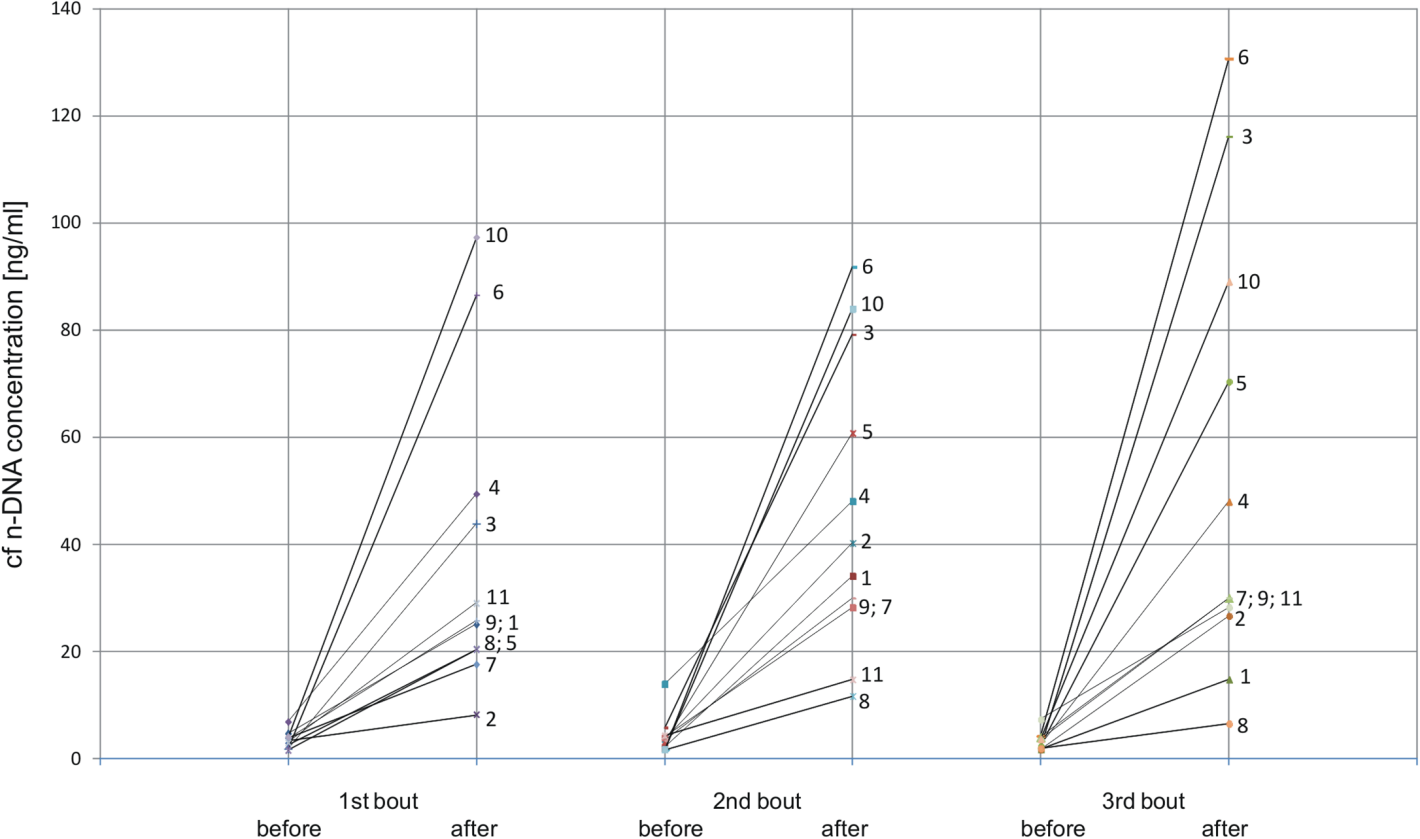
Individual cf n-DNA responses to each of three repeated bouts of exhaustive treadmill exercise separated by three days of resting. Eleven average-trained men executed three repeated treadmill exercise tests to exhaustion at speed corresponding to 70% of personal VO_2_ max and cf n-DNA was determined in pre- and post-exercise blood samples. The first studied men is marked by 1 and the last one is marked by 11. The same numbers at line segments represent responses of the same volunteer.

At baseline (before the first bout) mean plasma levels of cf n-DNA and cf mt-DNA were 3.4±1.4 ng/mL and 355±219*10^3^ GE/mL. Each bout of exhaustive exercise caused tremendous (more than 10-times) increase in mean plasma concentration of cf n-DNA (Table 4). Fig 2 shows individual responses of cf n-DNA to repeated bouts of exhaustive exercise. All studied volunteers (n = 11) revealed distinct increase in cf n-DNA after each bout of exercise. However, this response was transient and the elevated cf n-DNA completely returned to baseline values during 72 hours of resting between the first and the second bout as well as between the second and the third one (Table 4, Fig 2).

**Table 4:** Plasma concentrations of cell free nuclear (cf n-DNA) and mitochondrial (cf mt-DNA) DNA in eleven average-trained men before and after each of three bouts of exhaustive treadmill exercise.

Variable	Bouts of exhaustive treadmill exercise					
	1^st^ bout		2^nd^ bout		3^rd^ bout	
	before	after	before	after	before	After
cf n-DNA [ng/mL]	3.4±1.4 (3.3)	38.5±27.5* (25.8)	4.1±3.3 (3.4)	48.5±26.2* (45.2)	3.1±1.6 (2.4)	53.8±39.9* (32.0)
cf mt-DNA [*10^3^ GE/mL]	355±219 (294)	461±327 (360)	229±216† (174)	450±228* (416)	176± 120† (98)	462±314* (285)

Results are expressed as mean ± standard deviation (and median in parentheses). Other details as for Table 1.

* vs corresponding value before the bout, p<0.05.

† vs corresponding value before the first bout, p<0.05.

Although all bouts increased cf mt-DNA to almost the same level (around 460*10^3^ GE/mL), the significant difference (p<0.05) between pre- and post-exercise cf mt-DNA was noted only for the two last bouts (Table 4). This was due to the decrease in pre-exercise plasma concentration of cf mt-DNA only before the second and third bout of exercise. Mean cf mt-DNA concentration before the third bout was 2-times lower (p<0.05) than that before the first one (Table 4).

### Comparison of exhaustive exercise-induced changes in cell free nuclear DNA with alterations in other metabolic and muscle damage markers

Table 5 shows the percentage increments in measured variables caused by three repeated bouts of exhaustive exercise separated by 3 days of resting. The highest increment was noted for cf n-DNA and then for lactate. The consecutive responses (expressed as % increment) of cf n-DNA did not differ from each other although an upward trend was observed. The same was noted for other markers except for cf mt-DNA. In this case, the first response was lower than the second and third ones.

**Table 5:** Percentage increments in circulating cell free nuclear and mitochondrial DNA and selected metabolic and muscle damage markers in response to three repeated bouts of exhaustive treadmill exercise in healthy average-trained men.

Variable	Bouts of exhaustive treadmill exercise		
	1^st^ bout	2^nd^ bout	3^rd^ bout
cf n-DNA	1267±1185 (712)*	1705±1721 (1023)*	1896±1663 (1501)*
cf mt-DNA	70±117 (73)	327±342 (257)#	307±297 (209)#
Lactate	615±643 (351)†	655±612 (481)†	457±341 (561)†
CK	30±28 (30)	33±50 (30)	21±16 (17)
AST	27±28 (17)	21±27 (15)	46±111 (15)
ALT	18±21 (11)	14±27 (12)	33±82 (11)
Creatinine	39±19 (36)	36±19 (31)	26±18 (23)
Urea	10±6 (10)	11±8 (9)	10±10 (7)

cf n-DNA–cell free nuclear DNA, cf mt-DNA–cell free mitochondrial DNA, CK–creatine kinase, AST–asparate aminotransferase, ALT–alanine aminotransferase. Results are expressed as mean ±standard deviation (and median in parentheses). Other details as for Table 1.

* vs all corresponding values obtained for other variables, p<0.05.

† vs corresponding values obtained for CK, AST, ALT, creatinine and urea.

# vs corresponding percentage increment caused by the first bout, p<0.05

## Discussion

In this study we found that each of three repeated bouts of exhaustive treadmill exercise (separated by 3 days of resting) induced cf DNA response in healthy average-trained men. This response consisted on tremendous rise in circulating cf n-DNA as well as a moderate increase in cf mt-DNA and was accompanied by the rise of markers of skeletal muscles damage and metabolic reaction. Pre-exercise levels of cf n-DNA were stable, while pre-exercise cf mt-DNA gradually decreased over the study period. These findings suggest no development of tolerance to repeated bouts of exhaustive exercise as the factor causing the increase in circulating cf DNA levels. To our best knowledge this is the first study comparing cf DNA response to repeated bouts of exercise under laboratory conditions.

### Changes of plasma levels of cf n-DNA in response to repeated bouts of exhaustive exercise

We expected that rise in circulatory cf n-DNA caused by the repeated bouts of exhaustive exercise would decrease in order of appearance due to some adaptive processes and to the development of tolerance to these stimuli. Surprisingly, no decrease in cf n-DNA response was noted and even the mean percentage increase in cf n-DNA revealed an upward (although not significant) trend. Since the pre-exercise cf n-DNA levels were almost stable, one may conclude that each bout caused the release of the similar amount of n-DNA from cells and this phenomenon is transient and completely normalized during 3 days of resting between bouts. Mean concentration of cf n-DNA increased 12.7-, 17.1 and 18.9-times after the first, second, and third bout of exhaustive exercise, respectively. These results are compatible to previous observations showing rapid increase in circulating cf n-DNA in response to single bout of exercise of different type and load [7–14]. For instance, plasma cf n-DNA rose, in those previous studies, 14-times after treadmill run where velocity was increased by 2 km/h every 3min until exhaustion and 15-times after similar treadmill run till exhaustion [16,19]. Moreover, 10 km relay run induced the 7.6-fold increase in cf n-DNA levels while half marathon and ultramarathon caused the 18-fold difference between pre- and post-exercise cf n-DNA levels [11,12]. Some differences could result from different protocols, exercise load, and studied subjects. However, (what is important) the order of magnitude of exercise-induced cf n-DNA increase was comparable between these reports.

Increase in circulatory cf n-DNA occurred rapidly, within 9 min after the onset of incremental treadmill exercise test [22]. Consequently, cellular apoptosis and necrosis, leading to release of DNA fragments within hours [23,18], cannot be the main/only mechanism responsible for exercise-induced elevation of cf n-DNA in plasma. Therefore, the rise in circulating cf n-DNA after rapid strenuous exercise could be a consequence of DNA release from living cells [24,25] including DNA release from neutrophils extracellular traps [26]. Recent studies provided evidence of predominant contribution of DNA derived from hematopoietic cell lineage to baseline plasma cf n-DNA [27] and the exercise-induced rise in cf n-DNA [18].

Transient neutrophilia, monocytosis and lymphocytosis were reported after strenuous physical exercise [28,29]. In our study, each of three repeated bouts of exhaustive exercise induced the similar increase in these cells. The half-life of circulating neutrophils and monocytes is no longer than few days [30]. Neutrophils occur in two populations: a free-flowing pool present in blood and a marginated pool of cells. Physical exercise as well as adrenalin alone induced the movement of neutrophils from the marginated to the circulating pool [30,31]; however, they had no significant effect on the rate of neutrophils disappearance from the blood [31]. Little is known about mechanisms of neutrophils distribution between these pools. Moreover, it cannot be excluded that marginated and free-flowing neutrophils are functionally different [30], including DNA release in response to exercise. It is possible that during 3 days of resting period between bouts of exercise, important part of blood neutrophils and monocytes are replenished by newly formed cells and cause the similar cf n-DNA response to set of three repeated exhaustive exercises. However, this hypothesis and the precise determination of neutrophils and monocytes contribution to the increase in circulatory cf n-DNA induced by repeated exercises requires confirmation in further clinical studies.

### Changes of plasma levels of cf mt-DNA in response to repeated bouts of exhaustive exercise

Cell free mt-DNA rose after each of three repeated bouts of exhaustive exercise; however, the statistical significance was noted for the last two trials. In contrast, plasma concentration of pre-exercise cf mt-DNA declined over the study period by about 2-fold.

This is in line with previous studies reporting lack of significant increase in circulating cf mt-DNA just after single exhaustive exercise [16, 32]. On the other hand, prolonged moderate exercise (90 min treadmill run at 60% of VO2 max) caused the decrease in cf mt-DNA in healthy moderately trained young men [15]. In addition, professional male volleyball players presented lower circulating cf mt-DNA levels in comparison to non-athlete male volunteers [17]. These two afore mentioned reports on exercise–induced decline in cf mt-DNA are also consistent to our finding of gradual decrease in pre-exercise cf mt-DNA over the study period involving three bouts of exhaustive treadmill exercise. It should be pointed out that only in our study, in contrast to other investigations [15,16,32], a significant increase in post-exercise cf mt-DNA was found. This discrepancy may result from: (a) differences in the study protocols–we analyzed the effect of three repeated bouts of exhaustive treadmill exercise separated by 72 hours of resting while other researches described the effect of single bout of exercise on cf mt-DNA concentration; (b) more intense exercise performed by our volunteers that in terms of induction of cf n-DNA response could be equivalent to half-marathon [11] and may also stimulate increased release of cf mt-DNA.

However, significant increase in cf mt-DNA after the second and the third bout of exercise could, paradoxically, result from the exercise-induced decline in cf mt-DNA levels (what is in agreement with afore-mentioned studies). Post-exercise cf mt-DNA reached the same levels in all of the three bouts. Thus, lower pre-exercise cf mt-DNA concentration before the second and third bout caused a significant difference when compared with corresponding post-exercise levels.

Plasma levels of cf mt-DNA rose significantly after about 60 min of exhaustive exercise [11]. Thus, like in the case of cf n-DNA, it could not be a result of cell apoptosis or necrosis. It seems that release of mitochondrial DNA from living cells might be a responsible for this phenomenon. Although the cellular origin of these DNA fragments and mechanisms of their release are unknown, white blood cells could be the possible source since the increase was observed just after each bout of exhaustive exercise.

Viable human neutrophils released mt-DNA (with subsequent formation of neutrophil extracellular traps) after 35 min stimulation with granulocyte-macrophage colony-stimulating factor (GMCSF) and complement factor 5a (C5a) in vitro [33]. This process was not associated with apoptosis and necrosis [33] and, in addition, strenuous exercise increased circulating levels of C5a and GMCSF in healthy men [34,35]. Platelets contain functional mitochondria [36] that can be released after their activation [37]. Exhaustive exercise (e.g. marathon run) can induce platelets activation and their degranulation [38]. Although we did not analyze platelets function, their number increased by about 50 x10^3^ cells/μL after each bout of exercise. Thus, platelets could contribute to some extent to post-exercise rise in cf mt-DNA.

Pre-exercise cf mt-DNA levels decreased over the study period. This could be also the result of increased mt-DNA fragments clearance and inhibition of baseline mt-DNA release from cells.

Since pre-exercise levels of cf n-DNA were stable, it seems that unspecific clearance of cf mt-DNA from the plasma (e.g. liver and renal clearance) [39,40] could not be the main mechanism responsible for the decrease in cf mt-DNA concentration during the resting period between bouts. Apart from elevation of circulating cf n-DNA, exercise caused the rise in plasma DNAse activity [13]. Majority of cf n-DNA exists in complexes with histones while cf mt- DNA does not [41, 42]. Thus, cf mt-DNA could be more sensitive to degradation by DNAse [41,43] which may be responsible to some extent for gradual lowering of pre-exercise cf mt- DNA levels observed in our study. On the other hand, renal and visceral blood flow is transiently suppressed by about 40% during strenuous exercise in healthy men [44–46]. This can result in lower clearance of cf DNA from plasma and augment to some extent the elevating effect of exercise on the concentrations of circulating cf n-DNA and cf mt-DNA. Increased post-exercise plasma concentrations of urea and creatinine are in accordance with this hypothesis. However, exercise-induced catabolism can also contribute to the rise of these markers of renal function. It cannot be excluded that repeated strenuous exercise has a biphasic effect on mt-DNA release: at first, a direct rapid stimulation of mt-DNA release, and then inhibition of this process during the resting period. Nevertheless, it is still an open question whether this affects one or more types of cells and organs. This hypothesis can explain the decrease of elevated post-exercise cf mt-DNA levels to values lower than baseline (before the first bout); however, its confirmation requires further studies.

### Comparison of cf DNA response with other markers responses

In contrast to muscle injury and metabolic makers, cf n-DNA always rose after exhaustive exercise. Moreover, the mean percentage increase was several dozen-times higher than that found for the AST, ALT, CK, and creatinine. The second in the list after cf n-DNA was lactic acid; however, its percentage increase reached about half and one-fourth of that noted for cf n-DNA after the first and third bout of exercise, respectively. Although we did not investigate bouts of exercise with different load and intensity, it seems that cf n-DNA could be more sensitive for reflecting acute exercise-induced changes in human body than other markers used in our study. AST and ALT rose significantly only after the third bout of exhaustive exercise. The highest percentage increment of cf mt-DNA was observed also after the third bout. While ALT is a cytosolic enzyme, AST activity is found in cytoplasm as well as in the mitochondria. It is possible that simultaneous significant increase in AST and cf mt-DNA after the third bout could be the consequence of cellular processes leading to leakage of various biomolecules (including AST and mt-DNA) from mitochondria. However, further studies involving measurement of AST mitochondrial isoenzyme and cf mt-DNA in plasma are required to confirm this hypothesis.

### Strengths and weaknesses of the study

Strengths of this study were: (A) the unique protocol consisting of three exhaustive treadmill bouts separated by 3 days of resting and executed in a standardized laboratory environment; (B) simultaneous measurement of circulating cf n-DNA and cf mt-DNA accompanied by monitoring of metabolic and muscle damage markers; (C) careful recruitment of male volunteers that excluded any other clinical situations leading to alterations of cf DNA plasma levels. In addition, all involved volunteers were members of our university scientific or administrative staff and were therefore highly motivated to comply with instructions related to participation in the study. On the other hand, relatively low number of studied subjects (n = 11) could be recognized as a weakness of the study. Although the study group was sufficient to demonstrate significant changes of cf n-DNA and cf mt-DNA over the studied period, it was too little to calculate correlations between measured variables.

It should be pointed out that previous reports on effects of single bout of exercise on cf-DNA involved **a** numbers of volunteers similar to that studied in our trial. For instance, effect of ultramarathon [12] and weight lifting exercise [14] on cf-DNA was described in 14 and 12 athletes, respectively. Ten highly trained men were included for investigation of exercise-induced changes of circulating DNAse [13], and kinetics of post-exercise increase in cf-DNA was studied in a group of 11 men [19]. In addition, changes of cf mt-DNA related to seasonal training were described in 12 professional volleyball players [17]. The exercise-induced increase in cf-DNA was very high and this may explain the relatively low size of studied groups [11,12,14]. However, the low number of studied subjects cannot enable analysis and identification of major determinants of cf-DNA response to exercise, and it is clearly an important limitation of our study and the afore-mentioned ones. On the other hand, individual results of cf n-DNA responses (Fig 2) clearly show that all studied men revealed distinct increase in cf n-DNA after each bout of exhaustive exercise. This can prove that the observed effect of repeated bouts of exhaustive exercise on cf DNA is a true phenomenon regardless of the relatively low number of studied men.

Although the menstrual cycle seems to have no significant influence on the cf n-DNA in women [39], we did not include female volunteers in our study. This is the second limitation of our investigation. Menstrual cycle was reported to alter significantly the endurance performance in young women [40,47]. Female soccer players had lower maximal endurance performance in mid luteal phase [47]. Our study procedures lasted 13 days from VO_2_ max determination at the 1^st^ visit till the third exhaustive exercise test at the 4^th^ visit. Thus, for instance women in mid luteal phase at the study entry could finish the last visit in the follicular stage when endurance performance is higher. In addition, there are no data on effect of menstrual cycle on cf mt-DNA in women. That is why we decided to study cf DNA response to repeated bouts of exhaustive exercise only in men.

Mean run distance to exhaustion at the first bout of exercise was significantly shorter than those observed at the second and third bout. It could be recognized as some constraint of the study conclusion on stable (no tolerance development) cf n-DNA response to repeated exhaustive bouts of exercise in healthy men. However, while the mean run distance to exhaustion noted during the second and the third bout was the same, the cf n-DNA response to the third bout tended to be higher than the measured value at the second bout. Therefore, the afore-mentioned limitation could not lead to the biased conclusion of our investigation. It should be pointed out that run distance to exhaustion revealed great variability. We included to the study men who regularly performed recreational training where some of them practiced sport disciplines in the past. Therefore, our results could be applicable not only to professional athletes but to large population of average trained healthy men.

## Conclusions

We found that three repeated bouts of exhaustive exercise separated by three days of resting caused tremendous increase in circulating cf n-DNA in healthy moderately trained men with no signs of tolerance development. The exercise-induced increase in plasma cf mt-DNA was much lower than that of cf n-DNA and increased in order of appearance mainly due to lowered pre-exercise cf mt-DNA levels. Since cf n-DNA responses were many times-higher than those observed for typical markers of muscle injury and metabolism, measurement of circulating cf n-DNA could be a sensitive marker for monitoring acute exercise effects in human body. However, this conclusion needs confirmation in studies involving larger groups of volunteers of both sexes subjected to exercises with various loads and intensities executed both in laboratory and natural environment.

## Supporting information

S1 File Dataset. This file comprises the tables with determined individual results.
(XLSX)
